# Gene interaction enrichment and network analysis to identify dysregulated pathways and their interactions in complex diseases

**DOI:** 10.1186/1752-0509-6-65

**Published:** 2012-06-13

**Authors:** Yu Liu, Mehmet Koyutürk, Jill S Barnholtz-Sloan, Mark R Chance

**Affiliations:** 1 Center for Proteomics & Bioinformatics, Case Western Reserve University, Cleveland, OH, 44106, USA; 2 Department of Electrical Engineering & Computer Science, Case Western Reserve University, Cleveland, OH, 44106, USA; 3 Case Comprehensive Cancer Center, Case Western Reserve University, Cleveland, OH, 44106, USA; 4 Department of Epidemiology and Biostatistics, Case Comprehensive Cancer Center, Cleveland, OH, 44106, USA; 5 Department of Genetics, Case Western Reserve University, Cleveland, OH, 44106, USA

**Keywords:** Gene-gene interaction, Dysregulated pathways, Enrichment analysis, BAD pathway

## Abstract

**Background:**

The molecular behavior of biological systems can be described in terms of three fundamental components: (i) the physical entities, (ii) the interactions among these entities, and (iii) the dynamics of these entities and interactions. The mechanisms that drive complex disease can be productively viewed in the context of the perturbations of these components. One challenge in this regard is to identify the pathways altered in specific diseases. To address this challenge, Gene Set Enrichment Analysis (GSEA) and others have been developed, which focus on alterations of individual properties of the entities (such as gene expression). However, the dynamics of the interactions with respect to disease have been less well studied (i.e., properties of components ii and iii).

**Results:**

Here, we present a novel method called Gene Interaction Enrichment and Network Analysis (GIENA) to identify dysregulated gene interactions, i.e., pairs of genes whose *relationships* differ between disease and control. Four functions are defined to model the biologically relevant gene interactions of cooperation (sum of mRNA expression), competition (difference between mRNA expression), redundancy (maximum of expression), or dependency (minimum of expression) among the expression levels. The proposed framework identifies dysregulated interactions and pathways enriched in dysregulated interactions; points out interactions that are perturbed across pathways; and moreover, based on the biological annotation of each type of dysregulated interaction gives clues about the regulatory logic governing the systems level perturbation. We demonstrated the potential of GIENA using published datasets related to cancer.

**Conclusions:**

We showed that GIENA identifies dysregulated pathways that are missed by traditional enrichment methods based on the individual gene properties and that use of traditional methods combined with GIENA provides coverage of the largest number of relevant pathways. In addition, using the interactions detected by GIENA, specific gene networks both within and across pathways associated with the relevant phenotypes are constructed and analyzed.

## Background

Genome-wide mRNA expression data provide a rich resource for studying the molecular mechanisms of complex diseases. Through comparison of mRNA expression data between case and control samples, biomarkers and functional molecules significant for diagnosis, prognosis, and treatment have been identified for many complex diseases, including cancers [1,2]. Extracting signals while rejecting noise in the data and interpreting the results to elucidate biological mechanisms relevant to disease are however, challenging [3]. Lists of hundreds of mRNAs identified as differentially expressed are interesting but can be difficult to interpret in terms of the complex underlying biological processes. In addition, there are in many cases limited overlap between lists of individually dysregulated genes identified by different laboratories that study the same disease [3,4]. To overcome these challenges, a number of methods that consider genes not as individual entities but as members of biological relevant groups have been developed. Among such methods, gene set enrichment analysis (GSEA, [4]) is very powerful and highly popular.

While being quite useful for system-level analyses, GSEA and similar methods, such as gene set analysis (GSA, [5]) have a limitation: they focus only on the molecules (i.e., genes) that comprise a pathway and may neglect the changing interactions among genes within a pathway. Consequently, only pathways enriched in individual differentially expressed genes are detected with statistical significance. However, gene interactions and the dynamics of these interactions are also essential components of pathways and they underlie the orchestration of biological processes at many levels [6]. Interactions are associated with several dynamic characteristics, such as their direction, strength, permanence or transience, and presence or absence [6]. The biological influence of a pathway can be dramatically changed if the dynamics of the interactions in the pathway are altered. Indeed, several studies have demonstrated that the changes in the dynamics of interaction are associated with cancer and other diseases [7-9].

In this vein, Zhang et al. have proposed a method in which the interactions were represented by the co-variances or correlations between case and control classes, and showed that this approach provides biologically meaningful results [8]. Eddy et al. developed another method called DIfferential RAnk Conservation (DIRAC), which is based on the relative expression ranks of genes in a pathway [10,11]. A limitation of this method, however, is that it assesses the change in the relationship between genes qualitatively, and misses cases in which (i) changes in expression are not large enough to change the relative order of genes or (ii) the difference between the expressions levels becomes even larger. Watkinson et al. defined the synergy among pairs of genes in terms of the mutual information between phenotype and the clustering of samples induced by the gene expression levels [12] and extracted disease-specific interactions in cancer. Another class of algorithms for system-level analysis of differential gene expression aims to identify dysregulated subnetworks in disease [2]. Using protein-protein interaction (PPI) networks as a template for assessing functional associations among genes, these methods identify groups of functionally related genes that exhibit collective mRNA-level differential expression with respect to disease based on: mutual information, cover-based algorithms and others [13,14]. These results strongly suggest that dysregulation of interactions is as important a mechanism of disease as dysregulation of genes.

In order to further explore the dysregulation of gene interactions in disease, we have developed **G**ene **I**nteraction **E**nrichment and **N**etwork **A**nalysis (GIENA), which implements four mathematically simple, yet powerful interaction profile functions to model gene interactions. The hypothesis behind the analysis, suggested by the work described above, is that dysregulation of interactions, like the dysregulation of individual genes revealed by GSA, is an important set of variables to analyze to provide a comprehensive understanding of mechanisms of disease. GIENA attempts to provide a set of interaction profiles that are associated with universal biological concepts. We then use the canonical pathway information to drive a specific network analysis to indentify hub genes that may mediate communication across pathways. These profiles and their biological interpretation are as follows: (i) the sum of mRNA expression levels, which models cooperation, (ii) the difference between mRNA expression levels models competition, (iii) the maximum mRNA expression level models redundancy, and (iv) the minimum mRNA expression level models dependency between a pair of genes. This framework provides a basis for interrogating both the dynamics of multiple types of interactions and gives clues to the regulatory logic of the perturbed networks, both within pathways and across pathways, as opposed to simply identifying the dysregulated players.

We evaluated these four interaction profiles using previously published mRNA expression datasets associated with cancer [15-19]. We detected multiple disease-associated gene interactions, which we annotated with their biological significance and compared to known literature findings to validate the results. Also, we used the approach to compare data from different experimental studies to examine the robustness of the method. Then, we constructed gene interaction networks based on these detected interactions and analyzed the results as well, in this case to better understand potential novel connections *between* pathways and to provide testable hypothesis for future experimental validations. Our results show that GIENA is able to reliably detect both known and novel dysregulated canonical pathways and dysregulated interaction networks related to the disease. In addition, the method gives consistent results across datasets from disparate laboratories. Overall, GIENA is systematic approach for the identification of dysregulated interactions at the pathway level and provides specific guidance for interpretation of disease-specific interactions in complex diseases.

## Methods

### Models of gene interactions in GIENA

Four functions, named interaction profiles, are implemented to uncover different biological mechanisms that underlie the coordinated differential expression of the genes. *G* = {*g*_*1*_*, g*_*2*_*,…, g*_*n*_} denotes the set of genes for which mRNA expression data is available, *S =* {*s*_*1*_*, s*_*2*_*,…, s*_*m*_} denotes the set of samples, and *c*_*j*_ denotes the phenotype of sample *s*_*j*_*.* The normalized mRNA expression profile of gene *g*_*i,*_ is denoted by *m-*dimensional vector *e*_*i*_ such that *e*_*i*_(*j*) refers to the expression of gene *g*_*i*_ in sample *s*_*j*_ (*m* is the number of samples).

#### Cooperation (sum of expression)

Genes often cooperate with each other to perform various cellular functions and are organized into functional modules with densely connected genes within modules and a small number of interactions between modules [20]. Comparing the total expression across samples of interest can reveal disruptions in cooperative function. Indeed, Chuang et al. infer the activity of a subnetwork by averaging the normalized expression values of its member genes and identify dysregulated subnetworks in terms of the mutual information between this average expression and phenotype [13]. In our study, in order to systematically assess pairwise gene interactions, we use this concept in its simplest form: for each pair of genes, the sum of their mRNA expression levels is compared between disease and control samples to detect cooperation interactions dysregulated in diseases. Thus, we define the *cooperation profile* for genes *g*_*i*_ and *g*_*j*_ as

(1) $$t_{\mathrm{ij}}\left(k\right)=e_{i}\left(k\right)+e_{j}\left(k\right)\text{for}1\leq k\leq m$$

and quantify the strength of cooperative interaction between *g*_*i*_ and *g*_*j*_ in terms of the statistical significance of the difference of *t*_*ij*_ in disease and control samples.

#### Competition (difference in expression)

If two genes are working together to balance each other’s effects, assuming that their activities are correlated with mRNA expression, we can expect the difference between their mRNA expression levels to represent the regulatory balance between them. An example would include two transcription factors (TF) that act on a set of targets, but in opposite directions, i.e. one inhibits activity of the target promoter site while the other enhances activity. Consequently, changes in expression levels of these two TFs will result in maximal dysregulation of their targets when their abundance levels vary in *opposite* directions while their effects may be minimal when their abundance levels vary in the same direction. Motivated by these considerations, we define the *competition profile* of genes *g*_*i*_ and *g*_*j*,_ as

(2) $$d_{\mathrm{ij}}\left(k\right)=e_{i}\left(k\right)-e_{j}\left(k\right)\text{for}1\leq k\leq m$$

and quantify the strength of competitive interaction between *g*_*i*_ and *g*_*j*_.

Such comparisons of differences in mRNA expression levels have been used in disease classification. For example, the relative expression difference of *OBSCN* and *C9orf65* can distinguish two phenotypically similar cancers with high accuracy although the underlying biology is still unclear [21]. This method has been applied to construct gene regulatory networks and develop prognostic test for cancer [22,23]. Furthermore, Taylor et al. used difference in mRNA expression of the central hub gene in a subnetwork with its interacting partners to assess changes in the coherence of the subnetwork [24].

#### Redundancy (maximum expression) and Dependency (minimum expression)

Besides collectively working together, genes can cooperate in other ways, one example would be the wide-spread genetic interactions detected in yeast (deleting either of two genes has no obvious effect, removing both will have lethal effect [9]). For such pairs of genes, the suppression of both or over-expression of only one can be sufficient for dysfunction. To quantify its strength and detect gene interaction dysregulation in disease, we use the maximum mRNA expression for pairs of genes to define the *redundancy profile* of genes *g*_*i*_ and *g*_*j*_ as

(3) $$h_{\mathrm{ij}}\left(k\right)=\text{max}\left\{e_{i}\left(k\right),e_{j}\left(k\right)\right\}\text{for}1\leq k\leq m$$

In cases which two genes are required for a common function, suppression of one of them or over-expression of both may lead to dysfunction. To identify such interactions, we use the minimum mRNA expression for pairs of genes to define the *dependency profile* of genes *g*_*i*_ and *g*_*j*_ as

(4) $$l_{\mathrm{ij}}\left(k\right)=\text{min}\left\{e_{i}\left(k\right),e_{j}\left(k\right)\right\}\text{for}1\leq k\leq m$$

**Figure 1:**
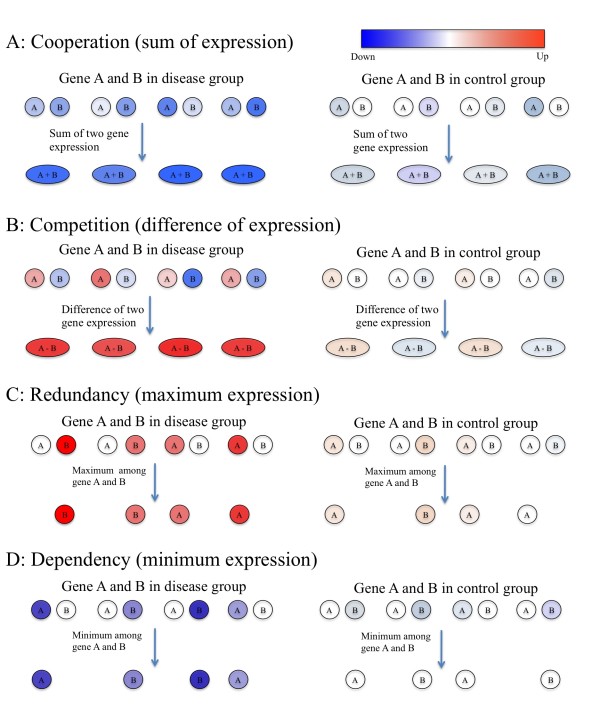
**Illustration of four genetic interaction profiles.** Note that in each case, the change of individual gene expression might not be statistically significant, but the change of the interaction profile could become statistically significant between case and control samples. A: cooperation profile. B: competition profile. C: redundancy profile. D: dependency profile.

These four gene interaction profiles are conceptually illustrated in Figure 1.

### Overview of GIENA

Using the four profiles described above for each pair of genes, we identify gene sets (pathways) that are enriched in dysregulated gene interactions, i.e.*,* pathways in which these profiles are significantly altered between disease and control samples for a large number of gene pairs in the pathway. For this purpose, we use the comprehensive pathway data downloaded from the Molecular Signatures Database (MSigDB) (http://www.broadinstitute.org/gsea/msigdb). We focus on the dataset that represents canonical representations of 633 biological processes compiled by domain experts (c2.cp, version2.5). Note that the genes in a pathway in MSigDB do not necessarily interact physically with each other; thus the interactions identified here are not to be confused with physical interactions and they should be considered as higher level “relationships”.

In order to identify the dysregulated pathways, GIENA uses the framework of GSA which generalizes the original GSEA [5]. For a pathway *P* with set of genes *g*_*1*_*, g*_*2*_*, …, g*_*k*_, the overall procedure is summarized as follows:

1) For each pair of *g*_*i*_ and *g*_*j*_ in *P,* the cooperation profile *t*_*ij*_, competition profile *d*_*ij*_, redundancy profile *h*_*ij*_, and dependency profile *l*_*ij*_ are calculated for disease and control groups separately. These four profiles are used to detect dysregulated pathways independently. In the following, we use the cooperation profile *t*_*ij*_ as an example to explain the procedure for detecting dysregulated pathways.

2) The classic two-sample *t*-statistic (*Z*_*ij*_) is calculated as the standardized difference of *t*_*ij*_ between disease and control groups. Repeating this procedure for each pair of genes in the pathway, a set of summary statistics *Z*(*P*) *=* {*Z*_*12*_*Z*_*13*_*… Z*_*1k*_*Z*_*23*_*Z*_*24*_*… Z*_*2k*_*… Z*_*k-1,k*_} is obtained for each pair of genes in the pathway. Note that, no hypothesis testing is done at this point; these statistics are only used to score the pathways.

3) The “maxmean” statistic S(P) for the pathway is computed to summarize *Z(P)*. The maxmean statistic is designed to detect unusually large z-values in either or both directions [5]. Namely, given the vector *Z*(*P*), the "maxmean" statistic is the mean of the positive or negative part of gene-pair scores in the pathway, whichever is larger in absolute value, i.e.:

(5) $$S\left(P\right)=\max\left\{{\sum_{Zij\in Z}{}^{+}}_{\left(P\right)}Z_{\mathrm{ij}}/{\left|Z^{+}\left(P\right)\right|}_{,}{\sum_{Zij\in Z}{}^{-}}_{\left(P\right)}Z_{\mathrm{ij}}/|Z^{-}\left(P\right)|\right\}$$

where *Z*^*+*^(*P*) *=* {*Z*_*ij*_*∈ Z*(*P*): *Z*_*ij*_ *>* 0} and *Z*^*-*^(*P*) *=* {*Z*_*ij*_*∈ Z*(*P*): *Z*_*ij*_ *<* 0}. It was shown previously that this statistic is more powerful than the modified Kolmogorov-Smirnov statistic used in the original GSEA [5].

4) *S(P)* is standardized by the mean and standard deviation of *t*_*ij*_ as in GSA. For details and the theoretical underpinnings of this procedure, please refer to [5].

5) The significance of *S(P)* is then evaluated using a permutation test. Namely, the data columns (sample labels) are permuted to generate a randomized dataset and this dataset is used to re-compute *S’.* Repeating this procedure for a sufficiently large number of times (B = 5000 permutations are performed in our experiments), a null distribution of standardized maxmean statistics S’_1_, S’_2_, …, S’_B_, is obtained. Using this distribution, the *p-value* for pathway *P* is estimated as the number of permuted datasets that yield a larger standardized maxmean statistic than the original dataset on *P,* i.e., *p-*value(P) = |{1 ≤ i ≤ B: S’_i_(P) ≥ S(P)}|/B.

6) Due to the stochastic nature of permutation test, *p*-values from each run will be slightly different (each single run has 5000 permutations). Thus, the permutation is repeated at least four times for each profile, and the average of the *p*-values is used.

7) In order to correct for multiple hypothesis testing in the procedure to detect dysregulated pathways, the *q*-value is calculated using the Q-value package [25]. Pathways with q-value ≤ 0.01 are considered significantly dysregulated.

Similarly, the above procedure is repeated for other interaction profiles, and enriched pathways are identified for each profile separately.

### Network construction

To construct the network enriched with dysregulated interactions, for each dyregulated pathway identified, each gene pairs are tested for dysregulation using classic *t*-test. To avoid the network with sparse and highly significant connections, a loose p-value threshold (0.05) without correction of multiple testing is applied.

### Gene expression data sets

#### P53 mutant data set

The National Cancer Institute (NCI) has collected a set of human cancer cell lines (NCI-60) derived from diverse tissues, like brain, blood, breast, and colon, etc. These cell lines have been subjected to various experiments including genotyping and gene expression analysis. Consequently, a wealth of genomic and validation data is available for the well-known tumor suppressor gene p53, which regulates the expression of a large number of genes in response to various signals of cellular stress and is often mutated in human cancers. For 50 of the NCI-60 cell lines, the p53 mutational status has been tested, and 17 are identified as wild type while the rest are mutant [15]. For these cell lines, the mRNA expression levels of 10,100 genes are also available [26] and downloaded from http://www.broadinstitute.org/gsea/data sets.jsp. In this study, these 50 cell lines are divided into two classes based on the status of p53 (wild type *vs* mutant), and GIENA and GSA methods are applied to detect pathways enriched in differential interactions and genes between two classes using the mRNA expression data.

#### Pancreatic cancer data set

Pancreatic cancer is often diagnosed at advanced stages. As a consequence, very few patients survive longer than five years after diagnosis. Ishikawa et al. compared the gene expression profiles of 24 pancreatic cancer patients and 25 healthy individuals to identify novel disease pathways [16]. We used this dataset (GSE1542) to identify the dysregulated pathways in pancreatic cancer.

#### Breast cancer datasets

GSA and GIENA were applied on three microarray datasets from previous studies to detect pathways associated with breast cancer staging and prognosis [17-19]. The datasets (GSE7390, GSE19615 and GSE26639) were divided into three groups based on the histological grading, and grades I and III were used for pathways detection. There are 30 grade I and 82 grade III tumors in GSE7390; 23 grade I and 64 grade III tumors in GSE19615 and GSE26639 contains 15 grade I and 121 grade III tumors. To make the latter dataset more balanced, 30 grade III tumors were randomly selected to compare with the 15 grade I tumors. GSA and GIENA were applied for each pair of grade I and III tumors respectively. The results from three datasets were compared to examine the reproducibility of the methods.

### Microarray data processing

Software Expander was used to process the microarray data [27]. The robust multichip average (RMA) and quantile normalization method were applied to normalize the data, and the expressions of multiple probesets are summarized to the expression of corresponding genes using Expander, then GIENA and traditional GAS were used to detect dysregulated pathways.

### Statistical testing of the overlap between physical and dysregulated interactions

In order to investigate the physical bases of the dysregulated interactions identified by GIENA, we compared these interactions with PPIs downloaded from a commonly used database Human Protein Reference Database, or HPRD. For each of the datasets used (p53, breast cancer, pancreatic cancer datasets), we separately identified the pairs of genes that (i) exhibit significantly dysregulated interactions and (ii) interact in the HPRD PPI network. We assessed the statistical significance of this overlap using hypergeometric test.

To be more precise, assume that r pathways are tested for a given dataset. For 1 ≤ i ≤ r, let c_i_ denote the number of pairs of genes in pathway i such that both genes in the pair has at least one interaction in HPRD. We use the following parameters for the hypergeometric test:

· $N=\sum_{i=1}^{r}c_{i}$: the number of gene pairs that are tested for dysregulated interaction and can potentially have a physical interaction (population size).

· *n:* the total number of significantly dysregulated interactions for the dataset of interest (sample size).

· *m:* the number of interactions in HPRD among proteins that together take part in at least one of the tested pathways, i.e., that have been tested for dysregulated interaction (total number of successes).

*k:* The number of gene pairs with a significantly dysregulated interactions and a physical interaction in HPRD (number of successes in the sample).

*Once N, n, m, and* k are obtained we compute the *p-value* of this observation as

(6) $$P(X>=k|N,n,m)=\sum \begin{array}{l} n\\ i=k \end{array}\frac{\left(\begin{array}{l} m\\ i \end{array}\right)\left(\begin{array}{l} N-m\\ n-i \end{array}\right)}{\left(\begin{array}{l} N\\ n \end{array}\right)}\text{,}$$

i.e., the probability that there would be at least k physical interactions among significantly dysregulated gene pairs if the dysregulated interactions were chosen at random. Here, X denotes the random variable that represents the overlap between the two sets of interactions. Note that we do not correct for multiple hypotheses since only one such test is performed for each dataset.

### Gene interaction network construction

Detected gene interactions are used to construct networks. These networks represent parts of the interactome which are disrupted in complex diseases. For each dysregulated pathway, interactions identified (with *p-value* <0.05) are collected. The network is generated and visualized using Cytoscape.

## Results and discussion

### GIENA reveals pathways and network dysregulated with respect to **p53 status in NCI-60 cell lines**

Enrichment results from GIENA and GSA for the p53 status data are shown in Table 1. GSA detects six pathways with q-values < 0.01. Two of them (p53 and p53 hypoxia) are directly linked to p53. Others have obvious links to tumorigenesis, such as the RAS pathway [28], which is also well understood to be related to p53 expression regulation [17]. The significant G1 & S phase pathway contains members that regulate the progression through G1-S phases of the cell cycle, such as CDK2 and CDK4 [29]. In the case of DNA damage, p53 accumulates in the cell and induces the inhibition of CDK [29]. This pathway also includes TP53, the protein product of p53, MDM2, the master regulator of p53 [30], and E2F, which regulates p53 indirectly [31].

**Table 1:** Q-values for pathways detected by of GIENA and GSA for the P53 dataset

**Pathway**	**Cooperation**	**Competition**	**Redundancy**	**Dependency**	**GSA**
P53	**0.005**	**0.004**	**0.007**	**0.006**	**<0.001**
P53 hypoxia	0.016	**0.004**	0.016	**0.006**	**<0.001**
G1 & S phase	0.035	**0.004**	0.037	**0.006**	**0.005**
EPONFKB	0.043	**0.004**	0.041	0.023	0.040
Mitochondria	0.043	**0.004**	0.041	0.021	0.032
BBCELL	0.016	**0.004**	0.013	0.024	0.040
BAD	0.016	**0.004**	0.011	0.021	0.019
RAS	0.016	0.047	0.020	**0.006**	**0.005**
ASBCELL	**0.005**	**0.005**	**0.005**	0.021	0.040
FAS signaling	0.016	**0.005**	0.041	0.024	0.032
ALS	0.043	**0.008**	0.041	0.024	0.032
RACCYCD	0.043	**0.008**	0.041	0.021	0.032
Programmed cell death	0.016	**0.009**	**0.009**	0.024	0.040
FML	0.045	0.05	0.046	0.026	**0.005**
HSP27	0.016	0.011	0.016	0.019	**0.007**

q-value < 0.01 is considered as significant, and highlighted in bold.

GIENA detects over twice as many pathways at q-values < 0.01 as compared to GSA (13 pathways with q-values < 0.01 vs. 6 for GSA), while missing two pathways detected by GSA, with four of the pathways, such as p53, p53 hypoxia, G1 & S phase and RAS, detected by both using the above q-value cutoff. These results suggest that mutations in p53 have profound affects at both individual gene and gene-gene interaction levels and that some of the pathways are primarily perturbed at the level of individual genes (seen by GSA alone), some are perturbed in both individual genes and in their interactions (intersecting pathways) and some are perturbed primarily at the level of interactions (seen by GIENA alone). Several pathways identified using GIENA alone are entirely confirmed by an examination of the literature. For example, the mitochondria pathway (role of mitochondria in apoptotic signaling), BAD (Regulation pathway of BAD phosphorylation), and FAS pathways [32-34] are all linked to apoptosis and highly relevant to p53 functions [35]. The BAD pathway is ranked relatively highly in the results from GSA, (eighth ranked pathway with q around 0.02), while it is assigned 5-fold more significant q-value*s* by GIENA (0.004) based on the competition profile. BAD exhibits dysregulation at the level of both the individual gene and at the level of gene interactions and GIENA can pinpoint relevant regulatory logic of the pathway that is potentially perturbed (see below). Specifically, these observations provide a testable hypothesis that a subset of competing interactions within the BAD pathway is critical to the changes seen as a result of p53 status.

**Figure 2:**
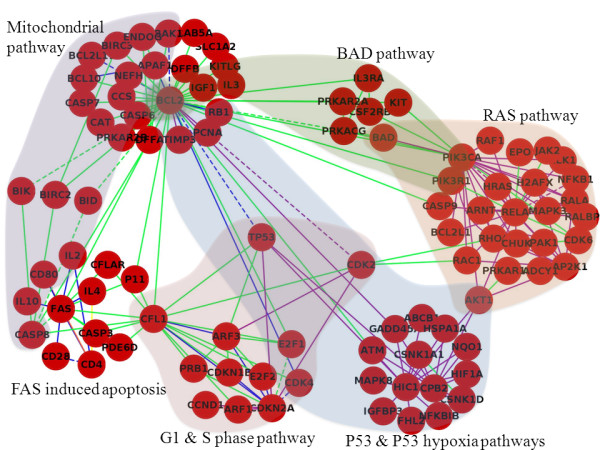
**Network for P53 dataset using GIENA.** Network generated using the dysregulated interactions identified by GIENA on the P53 dataset after filtering the significantly differentially expressed genes between cases and controls, and the resulting singletons. The dashed lines indicate that physical interactions between the products of the respective genes are reported in HPRD. The green lines represent competition interactions; purple lines represent dependency interactions; orange lines represent redundancy interactions. The blue lines indicate that these interactions were detected by multiple interaction profiles. Note that there is no cooperation interaction presented in this network, because no unique pathway is detected by cooperation profile.

In order to leverages the pathway results to uncover potential interesting interactions *across pathways,* we constructed a network of dysregulated interactions in which the edges represent dysregulated interactions from any of the four profiles. To simplify the network and focus on the novel findings from GIENA, genes that are significantly differentially expressed between cases and controls at q-values < 0.01, (in total three genes BAX, MDM2, and CDKN1A) are removed. Also, the 17 genes that did not have any significantly dysregulated interactions with the remaining nodes were also deleted. The sub-network after filtering is shown in Figure 2, which has 95 nodes with 186 interactions derived from six pathways and is organized to show the underlying relevant pathways based on data from MSigDB. The network in Figure 2 illustrates several typical characteristics of biological networks, such as the existence of hubs. There are hubs clearly located within pathway gene sets (e.g. FAS in FAS induced apoptosis and BCL2 in the mitochondrial pathway) as well as hubs connecting multiple pathways. For example, TP53 (the protein product of p53 gene), CFL1 (cofilin 1), and CDK2 (cyclin dependent kinase 2) exhibit significantly dysregulated interactions across at least three pathways. Although TP53 and CDK2 are not hubs within any particular pathway set, they directly link three and four dysregulated pathways, respectively. Nodes that connect more than one major regulatory module (pathway) we term "cross pathway" hubs. Interestingly some hubs exhibit only moderate differential expression between case and control (e.g., CFL1 has q-value: 0.03; FAS, 0.13; and PIK3CA, 0.14), i.e., the dysregulation of these individual genes is not captured by methods that are based on differential expression of individual genes alone. However, the interactions of these genes with other genes are disrupted in the phenotype, which is detected by GIENA. Cofilin 1 is an important regulator of the actin cytoskeleton and thus is a critical regulator of cell motility while CDK2 functions to signal the G1/S cell cycle transition. The GINEA analysis indicates that changes in the interaction of these proteins are important to the phenotypic differences relevant to p53 status. Overall, both within pathway hubs and cross pathway hubs are interesting candidates for experimental perturbation by knockdown or knockout, such experiments could define the relationship of the hub to overall phenotype and test the importance of the detected interactions. This is a stated purpose of the tool, to identify specific points of perturbation within pathways for experimental testing.

### Regulatory logic of BAD pathway probed by GIENA

**Figure 3:**
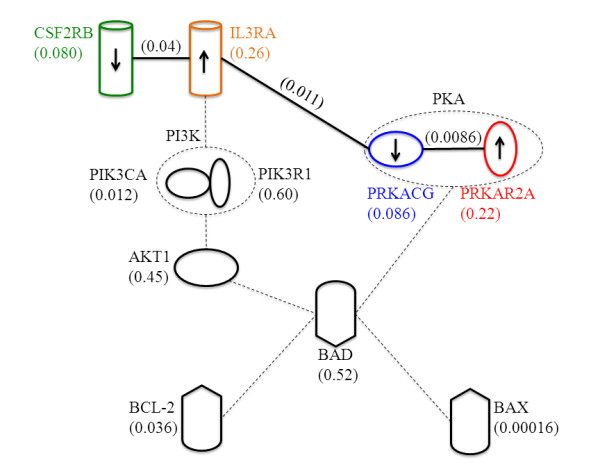
**Simplified BAD pathway.** Solid lines represent gene interactions detected by competition profiles using mRNA expression. Dashed lines indicate the physical interactions. The numbers beneath the gene names are p-values corresponding to the expression change between mutant p53 and wild type p53 samples. The numbers above lines are p-values for the change in the competition profile for two genes connected between mutant p53 and wild type p53 samples. The arrows inside the gene symbols indicate the trends of the mRNA expression of mutant p53 samples in respect to p53 wild type samples, though the change is not statistically significant.

In an attempt to explore the biological significance of the network connections suggested by GIENA, we examined the details of the regulation of BAD pathway. Figure 3 shows a simplified BAD pathway that includes: two genes that show dysregulation with respect to individual gene expression in the p53 datasets (e.g. BAX and PIK3CA), three pairs of genes that show dysregulated interactions (CSF2RB-IL3RA, IL3RA-PRKACG, and PRKACG-PRKAR2A), and additional genes that connect them with BAD as the hub.

The dysregulation of the genes BAX and PIK3CA provided a q-value of 0.02 in the GSA based analysis of this pathway while the competition profile from GIENA generated greater significance with a q-value of 0.004 (Table 1). Thus, an examination of interactions provides greater confidence in establishing dysregulation of this pathway than examining dysregulated genes alone. In examining the individual gene *p-values*, we can see that neither CSF2RB, IL3RA, PRKACG, nor PRKAR2A are dysregulated at the individual gene level, their interactions that show significant changes between phenotype and control (Figure 3). Detailed inspection of the expression patterns of these genes shows that CSF2RB is slightly (but not significantly, *p-value* 0.08) down regulated in case vs. control while IL3RA is slightly up-regulated (but not significantly, *p-value* 0.26). IL3RA gene encodes the interleukin 3 specific ligand binding subunit of a receptor hetero-dimer complex where the signaling domain is shared among and responds to multiple ligands, including colony stimulating factor 2. Thus, we suggest that the reciprocal expression changes in the CSF2RB-IL3RA pair provide a finely tuned system for maintaining molecular balance in downstream signaling to PI3K, and subsequently to AKT1 and BAD, which can provide tight control for apoptosis signaling overall. This concept of molecular balance has been previously elaborated for PI3K signaling [36]. Note that the competition profile also reveals potential regulation by molecular balance in the PRKACG-PRKAR2A (cyclic AMP dependent protein kinase gamma catalytic subunit and type-II alpha regulatory subunit) ligand-receptor interactions as well. Thus, the use of the competition profile revealed sub-components of the BAD pathway that are involved in maintaining tight molecular balance of signaling, changes that could not be detected by individual gene expression alone.

### GIENA discovers dysregulated pathways and networks in pancreatic cancer

Enrichment results from GSA and GIENA for the pancreatic cancer data are shown on Table 2. GSA does not detect any pathway with a significant q-value. In comparison, GIENA detects nine pathways, including glycosphingolipid biosynthesis, ACE2 (angiotensin-converting enzyme 2), and several complement pathways. Some of these pathways were previously shown to be related to cancer but not much is known about them in pancreatic cancer. Complement pathways are related to cell killing, recent studies have shown that an activated complement pathway can kill tumor cells [37], thus, its association with pancreatic cancer is logical. The angiotensin converting enzyme precursor 2 (ACE2) pathway is top ranked with a q-value 0.004; ACE2 protein is a component of the renin-angiotensin-aldosterone system (RAAS), which regulates blood pressure and water (fluid) balance. Recent studies show that ACE2 is down-regulated in some cancers [38]. In terms of other significant pathways accumulation of glycosphingolipid has been observed in cancer cells [39] and it has been shown that activated complement pathways can kill tumor cells [37]. These results suggest that the alterations in the expression of single genes are often subtle in pancreatic cancer and these pathway alterations can be captured only when interactions are considered.

**Table 2:** Q-values of pathways detected by GIENA for the pancreatic cancer dataset

**Pathway**	**Cooperation**	**Competition**	**Redundancy**	**Dependency**	**GSA**
Glycosphingolipid biosynthesis	0.018	**0.002**	**0.004**	0.027	0.064
Classic complement activation	0.047	**0.003**	0.040	0.027	0.052
Classic	0.047	**0.003**	0.034	0.027	0.064
Classic & alternative complement	0.047	**0.004**	0.039	0.026	0.052
ACE2	0.047	**0.004**	0.027	0.026	0.064
Bisphenol A degradation	0.049	**0.009**	0.048	0.027	0.064
Lysine degradation	0.037	**0.009**	0.040	0.028	0.061
Renin-angiotensin system	0.047	**0.009**	0.035	0.027	0.052
Glycosphingolipid biosynthesis (neo-lactoseries)	0.018	0.026	**0.004**	0.027	0.064

q-value < 0.01 is considered as significant, and highlighted in bold. Note none of pathways has significant q-value using GSA.

**Figure 4:**
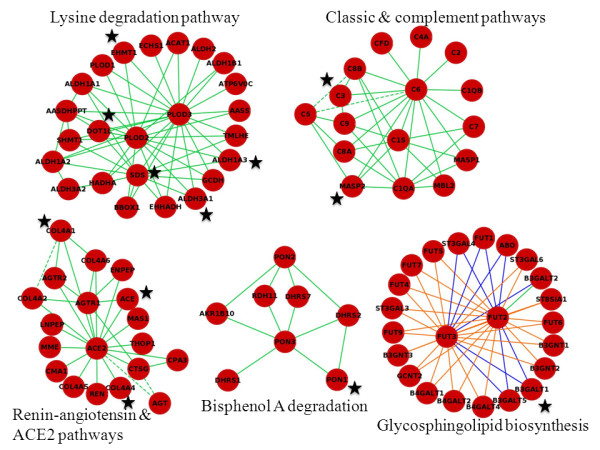
**Network generated using the dysregulated interactions identified by GIENA on the pancreatic cancer dataset.** Note that no genes were identified as differentially expressed between pancreatic cancer and normal cells. The dashed lines indicate that physical interactions between the products of the respective genes are reported in HPRD. See Figure 2 legend for color coding of interactions. The mutated genes detected by Jones et al. are marked by a star [40].

The network generated using the dysregulated interactions detected by GIENA on the pancreatic cancer dataset is shown in Figure 4. Note that there is no significantly differentially expressed gene (0.01 q-value level). The network has five separate pathways without identification of cross pathway hubs involved across the disparate pathways. It may be that multiple unlinked pathways are dysregulated in pancreatic cancer or that important cross pathways hubs proteins are as yet unidentified due to limited coverage in interaction databases. However, within the five pathways several hub genes are identified, such as, fucosyl transferase 3 (FUT3), Procollagen-lysine 2-oxoglutarate 5-dioxygenase 3 (PLOD3), paraoxanase 3 (PON3) and ACE2 and these are interesting targets for further experimental investigation in the disease.

To confirm the GIENA findings, the results are compared with that of Jones, et al., which identified genes mutated in pancreatic cancer by genome-wide protein-coding-gene sequencing of 24 patients [40]. Comparison of this data to our pancreatic cancer networks shows that the dysregulated networks identified by GIENA contain 12 mutated genes, and each network has at least one mutated gene (Figure 4, mutated genes are marked with a star). In particular, five mutated genes are present in the lysine degradation network including: two aldehyde dehydrogenases (ALDH1A3 and ALDH3A1), DOT1-like histone H3 methyltransferase (DOT1L), euchromatic histone-lysine N-methyltransferase 1 (EHMT1), and serine dehydratase (SDS). More interestingly, although there is no evidence of physical interaction between them in HPRD (Human Protein Reference Database), GIENA suggests that SDS interacts with ALDH1A3, ALDH3A1 and DOT1L (Figure 4). Thus, the pathways detected by GIENA are supported by recent mutation data. In addition, the epistatic effects of the mutations are predicted by the GINEA framework.

### Pathways associated with breast cancer prognosis are consistent across datasets

Breast cancer prognosis is largely driven by the assessment of key clinical characteristics, such as tubule formation, mitotic rate, and nuclear pleomorphism [41]; these are generally combined in a clinical grade. This approach has become a widely used strategy in the assessing risk of disease relapse and estimating the benefit of a treatment strategy. More recently, genomic profiling combined with clinical information was used to refine prognosis and improve therapeutic strategies for breast cancer [42]. As outlined in the methods section, we have identified gene expression data sets nominally associated with stages I and III. To identify pathways that vary between the relevant stages, GSA and GIENA were applied to three previously published datasets [17-19]. GIENA detected 11 pathways with significant q-values in at least two datasets (Table 3). Most are clearly associated with tumorigenesis and development including changes in interactions of SRC (oncogene) and RB (suppressor) pathways. Overall, more than half of the detected pathways are related to cell cycle (such as cell cycle, P27, Cyclin, CDC25, G2 and M phase transition, and G2 pathways) and are significantly dysregulated for grades I vs. III. Of these 11 pathways all but two were detected by GSA (Table 4). The two pathways missed by GSA are FBW7 and P27 pathways, FBW7 is a well-known tumor suppressor and P27 is associated with breast tumor prognosis [43,44]. Moreover, both pathways are ranked in top 25 pathways of GSA results for all three datasets, but with q-values below the threshold, and all pathways detected by GSA are also detected by GIENA. These results suggest that in breast cancer most pathways are dysregulated at both the individual gene and at the level of interaction.

**Table 3:** Q-values of pathways detected by GIENA for three breast cancer datasets

**Pathways**	**Cooperation**			**Competition**			**Redundancy**			**Dependency**		
	Dataset 1	Dataset 2	Dataset 3	Dataset 1	Dataset 2	Dataset 3	Dataset 1	Dataset 2	Dataset 3	Dataset 1	Dataset 2	Dataset 3
***P27***	0.027	0.021	0.0073	0.019	0.025	0.016	0.023	0.038	0.019	**0.014**	**0.010**	**0.0062**
***FBW7***	0.018	0.021	0.0071	0.019	0.028	0.016	0.021	0.030	0.019	**0.0062**	**0.020**	**0.010**
PTC1	**0.018**	**0.0073**	**0.010**	**<0.0001**	**0.0013**	**0.014**	0.012	0.013	0.019	0.0097	0.015	0.025
Cell cycle	0.018	0.018	0.010	0.0082	0.021	0.014	0.021	0.032	0.019	**0.0043**	**0.0020**	**0.017**
SRCRPTP	**0.0021**	**<0.0001**	**0.0063**	**0.0022**	**0.0074**	**0.016**	**0.0018**	**0.002**	**0.0036**	**<0.0001**	**0.0010**	**0.012**
G2 &M phase	**0.0095**	**0.0056**	**0.010**	**0.0015**	**0.0033**	**0.014**	0.014	0.020	0.019	**0.0017**	**0.0010**	**0.017**
Cyclin	0.018	0.015	0.010	0.0017	0.021	0.016	0.021	0.029	0.018	**0.0021**	**0.0010**	**0.010**
RB	**0.0095**	**0.0078**	**0.0071**	**0.0081**	**0.0092**	**0.016**	0.014	0.020	0.018	**0.0017**	**0.0023**	**0.017**
G2	**0.0021**	**0.0056**	**0.010**	0.0082	0.016	0.016	0.0018	0.013	0.019	**0.0014**	**0.0023**	**0.020**
SKP2E2F	0.015	0.018	0.0071	0.019	0.029	0.016	0.019	0.025	0.018	**0.0043**	**0.015**	**0.0086**
CDC25	**0.0035**	**0.0033**	**0.0073**	0.0082	0.016	0.016	0.0093	0.013	0.018	**<0.0001**	**0.0010**	**0.017**

q-value < 0.01 is considered as significant, and q-values for pathways with significant q-values for at least two datasets are highlighted in bold.

Two pathways detected by GIENA only are in italics.

Dataset 1: GSE7390; Dataset 2: GSE19615; Dataset 3: GSE26639.

Only pathways detected by at least two datasets listed.

Both P27 and FBW7 pathways are in top 25 pathways in normal GSA results in all three datasets.

**Table 4:** Q-values of pathways detected by GSA for the three breast cancer datasets

	**Dataset 1**	**Dataset 2**	**Dataset 3**
PTC1	0.0077	0.0072	0.013
Cell cycle	<0.0001	0.00027	0.0072
SRCRPTP	0.0020	0.0027	0.0043
G2 &M phase	0.0048	0.0025	0.0075
Cyclin	0.019	0.0072	0.0050
RB	0.0048	0.0030	0.0072
G2	0.0020	0.00027	0.011
SKP2E2F	0.0069	0.012	0.0070
CDC25	0.0022	0.00027	0.0075

q-value < 0.01 is considered as significant.

Only pathways detected by at least two datasets are listed.

Dataset 1: GSE7390; Dataset 2: GSE19615; Dataset 3: GSE26639.

**Figure 5:**
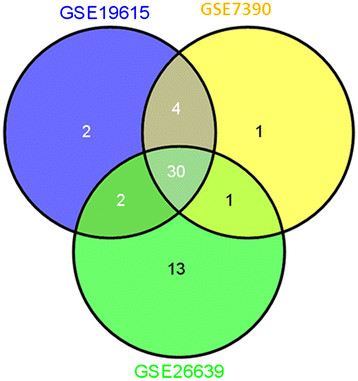
**Venn diagram of comparison of detected gene interactions.** To test the reproducibility of GIENA, the detected interactions for P27 pathway are pair-wisely compared for three breast cancer datasets. Majority of the interactions are detected in all three datasets. Especially, more than 90% of interactions are shared between GSE19615 and GSE7390.

Microarray data are often noisy, and consequently, the reproducibility often is low across datasets from different laboratories for the same disease. We further examined the consistency of the pathways detected by GIENA across three datasets. In total, 22 pathways are assigned significant q-values for at least one dataset (data not shown) and 11 of 22 are significant for at least two datasets (Table 3), while the others are often ranked in the top 50 pathways. We also examined the consistency of gene interactions detected by GIENA. For P27 pathway identified by GIENA only, we investigated the overlap of gene interactions among three datasets. Results show that 65-83% interactions are shared among all three datasets (Figure 5), and a pairwise comparison between GSE7396 and GSE19615 shows even higher overlap; more than 92% of interactions are shared. Similar overlap is observed for FBW7 pathway, which is also detected by GIENA, but not GSA. It should be noted that results from dataset GSE26639 is most dissimilar from the other two, possibly due to its small sample size (it has the smallest number of grade I patients). In summary, GIENA results are robust and consistent across different datasets in identification of both gene interactions and pathways and provide results consistent with the literature.

### Comparison of interaction profiles detected in different pathways

In order to investigate the biological relevance of the four proposed interaction profiles (cooperation, competition, redundancy and dependency), we compared enrichment results for the four profiles. The comparison shows that the detected pathways are different among most of the four profiles in many cases (the exception is cooperation and redundancy, see below), which might reflect the various underlying biological processes of complex diseases, e.g., in some conditions the genes compete to influence phenotype; in others, cooperation might drive dysregulation.

**Figure 6:**
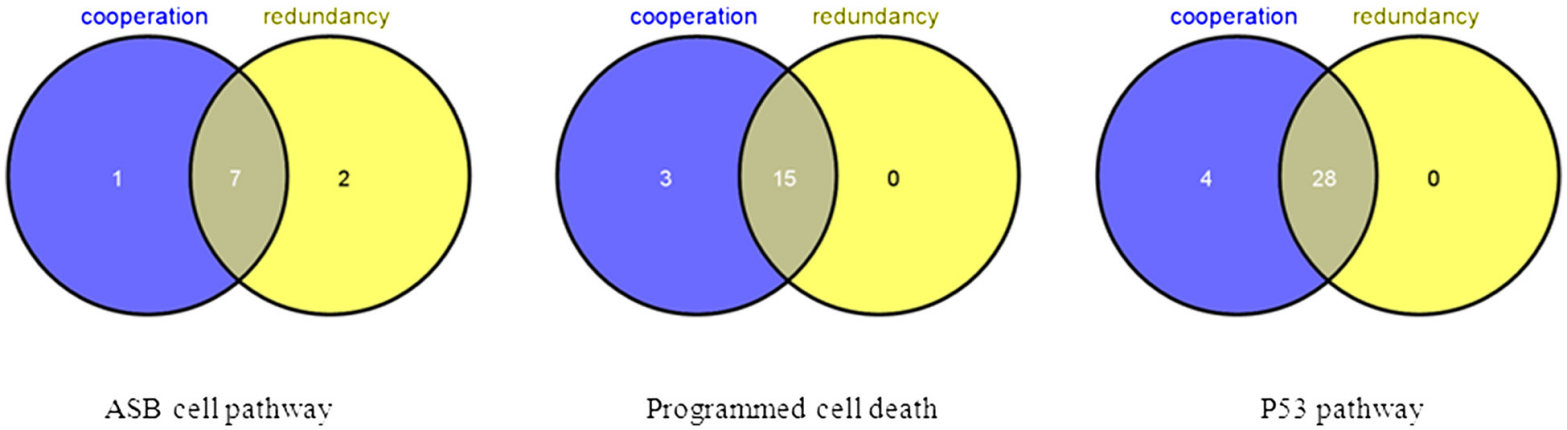
**Venn diagram of comparison of detected cooperation and redundancy interactions.** Pathways detected by both profiles are similar (Table 1); the comparison of detected interactions also shows high level of similarity.

Pathways detected by cooperation (sum) and redundancy (higher) profiles are similar in the results from the p53 dataset, e.g. the p53, ABSCELL, and programmed cell death pathways are identified by both approaches. In fact many gene interactions from these two profiles are significant for these pathways (Figure 6). This is not surprising, since if the expression of one of the genes involved in the interaction changes dramatically, and the expression of this gene is much higher than the other gene, then the sum and higher expression of the two genes will converge to each other. The competition profile has a strong influence on the identified pathways, as seen in Figure 2 and 4 (green line represents interactions detected by competition profile). The reason is not obvious, and further investigation is needed to reveal it.

It should be noted that the four profiles are related, for example, the absolute difference equal to the difference of maximum and minimum profiles. However, the information on the directionality would be missed if difference were replaced by absolute difference. To further investigate the performance of four profiles, we investigated the number of overlapping pathways detected by two profiles in three breast cancer datasets. The results from three datasets are highly similar; table 5 lists the results from dataset 1 (GSE7390). Overall, three profiles (cooperation, competition, and dependency) contribute to the identification of dysregulated pathways in breast cancer datasets. Although all pathways detected by redundancy profile are identified by other profiles in breast cancer cases, it did identify one unique pathway in pancreatic cancer dataset (Glycosphingolipid biosynthesis, table 2). Therefore it is useful to consider all four profiles to comprehensively identify significantly dysregulated pathways due to the high heterogeneity of cancer datasets.

**Table 5:** Comparison of performance of four profiles in dataset 1 (GSE7390) of breast cancer

	**Cooperation**	**Competition**	**Redundancy**	**Dependency**
Cooperation	-	5/5	3/5	5/5
Competition	5/8	-	3/8	8/8
Redundancy	3/3	3/3	-	3/3
Dependency	5/10	8/10	3/10	-

The listed are number of overlapping pathways detected by two profiles divided by the number of pathways detected by the profile in each row. For other two datasets of breast cancer, the results are similar.

### Nature of detected interactions

It has been repeatedly shown that human diseases are associated with perturbations of physical PPIs. In order to investigate the nature of the dysregulated interactions identified by GIENA, we compare these interactions with physical PPIs downloaded from HPRD. The results show that the overlap between PPI and detected gene interactions are significant in the p53 dataset: among 215 detected gene interactions in p53 dataset, 23 pairs also physically interact with each other in a network of PPIs (*p-value* < 1.2 × 10^-4^). In the case of the pancreatic cancer dataset, 5 out of 173 gene pairs have physical interaction in HPRD (*p-value* < 0.13). This observation suggests that, while a significant number of dysregulated interactions stem from physical interactions, the nature of many gene interactions may be indirect and mediated by other genes, or their interactions are not discovered by current experiments due to the overall low coverage of the interactome in HPRD.

## Conclusions

In summary, GIENA generalizes the gene-based enrichment method to detect pathways that are dysregulated in diseases based on changes in multiple types of interactions. Three datasets are used to demonstrate its potential; the results reveal several well-known and biologically meaningful pathways associated with cancer; and the results are highly reproducible. Comparison with GSA indicates that our method is comprehensive and efficient in terms of extracting weak signals and identifying pathways that are statistically significant but that a combination of GSA with GIENA provides the most comprehensive survey of pathway level dysregulation.

## Abbreviations

GSEA, Gene Set Enrichment Analysis; GSA, Gene Set Analysis; GIENA, Gene Interaction Enrichment and Network Analysis; HPRD, Human Protein Reference Database.

## Competing interests

The authors declare that they have no competing interests.

## Authors’ contributions

YL designed and performed the study and drafted the manuscript. MK participated in the design of the study and helped to draft the manuscript; JSB-S participated in the design of the statistical testing; MRC designed the study and drafted the manuscript. All authors have read and approved the final manuscript.

## References

[B1] Rhodes DR Yu J Shanker K Deshpande N Varambally R Ghosh D Barrette T Pandey A Chinnaiyan AM ONCOMINE: a cancer microarray database and integrated data-mining platform Neoplasia (New York, NY) 2004 6 1 6 10.1016/s1476-5586(04)80047-2 PMC1635162 15068665

[B2] Liu Y Patel S Nibbe R Maxwell S Chowdhury SA Koyuturk M Zhu X Larkin EK Buxbaum SG Punjabi NM Russ B, Altman A, Keith D, Lawrence H, Teri E Systems biology analyses of gene expression and genome wide association study data in obstructive sleep apnea Proceedings of The Pacific Symposium on Biocomputing (PSB) 2011: 3-7 Jan., 2011; Big Island of Hawaii 2011 Klein: electronic proceedings, 14 23 10.1142/9789814335058_0003 PMC4465214 21121029

[B3] de la Fuente A From 'differential expression' to 'differential networking' - identification of dysfunctional regulatory networks in diseases Trends in genetics : TIG 2010 26 326 333 10.1016/j.tig.2010.05.001 20570387

[B4] Subramanian A Tamayo P Mootha VK Mukherjee S Ebert BL Gillette M Paulovich A Pomeroy SL Golub TR Lander ES Mesirov JP Gene set enrichment analysis: a knowledge-based approach for interpreting genome-wide expression profiles Proc Natl Acad Sci U S A 2005 102 15545 15550 10.1073/pnas.0506580102 16199517 PMC1239896

[B5] Efron B Tibshirani R On testing the significance of sets of genes Annals of Applied Statistics 2007 1 107 129 10.1214/07-AOAS101

[B6] Beyer A Bandyopadhyay S Ideker T Integrating physical and genetic maps: from genomes to interaction networks Nat Rev Genet 2007 8 699 710 10.1038/nrg2144 17703239 PMC2811081

[B7] Marchini J Donnelly P Cardon LR Genome-wide strategies for detecting multiple loci that influence complex diseases Nat Genet 2005 37 413 417 10.1038/ng1537 15793588

[B8] Zhang J Li J Deng H-W Identifying gene interaction enrichment for gene expression data PLoS One 2009 4 e8064 10.1371/journal.pone.0008064 19956614 PMC2779493

[B9] Costanzo M Baryshnikova A Bellay J Kim Y Spear ED Sevier CS Ding H Koh JL Toufighi K Mostafavi S The genetic landscape of a cell Science 2010 327 425 431 10.1126/science.1180823 20093466 PMC5600254

[B10] Eddy J Geman D Price ND Relative expression analysis for identifying perturbed pathways Conference proceedings : Annual International Conference of the IEEE Engineering in Medicine and Biology Society IEEE Engineering in Medicine and Biology Society Conference 2009 2009 5456 5459 19964680 10.1109/IEMBS.2009.5334063 PMC2923586

[B11] James E Hood L Price ND Geman D Identifying Tightly Regulated and Variably Expressed Networks by Differential Rank Conservation (DIRAC) PLoS Comput Biol 2010 6 e1000792 10.1371/journal.pcbi.1000792 20523739 PMC2877722

[B12] Watkinson J Wang X Zheng T Anastassiou D Identification of gene interactions associated with disease from gene expression data using synergy networks BMC Syst Biol 2008 2 10 10.1186/1752-0509-2-10 18234101 PMC2258206

[B13] Chuang HY Lee E Liu YT Lee D Ideker T Network-based classification of breast cancer metastasis Mol Syst Biol 2007 3 140 17940530 10.1038/msb4100180 PMC2063581

[B14] Chowdhury SA Nibbe RK Chance MR MK Subnetwork state functions define dysregulated subnetworks in cancer J Comput Biol 2011 in press 10.1089/cmb.2010.0269 PMC3123978 21385033

[B15] Olivier M Eeles R Hollstein M Khan MA Harris CC Hainaut P The IARC TP53 database: new online mutation analysis and recommendations to users Hum Mutat 2002 19 607 614 10.1002/humu.10081 12007217

[B16] Ishikawa M Yoshida K Yamashita Y Ota J Takada S Kisanuki H Koinuma K Choi YL Kaneda R Iwao T Experimental trial for diagnosis of pancreatic ductal carcinoma based on gene expression profiles of pancreatic ductal cells Cancer Sci 2005 96 387 393 10.1111/j.1349-7006.2005.00064.x 16053509 PMC11160054

[B17] Li JY Paragas N Ned RM Qiu A Viltard M Leete T Drexler IR Chen X Sanna-Cherchi S Mohammed F Scara5 is a ferritin receptor mediating non-transferrin iron delivery Dev Cell 2009 16 35 46 10.1016/j.devcel.2008.12.002 19154717 PMC2652503

[B18] Li Y Zou L Li Q Haibe-Kains B Tian R Desmedt C Sotiriou C Szallasi Z Iglehart JD Richardson AL Wang ZC Amplification of LAPTM4B and YWHAZ contributes to chemotherapy resistance and recurrence of breast cancer Nat Med 2010 16 214 218 10.1038/nm.2090 20098429 PMC2826790

[B19] de Cremoux P Valet F Gentien D Lehmann-Che J Scott V Tran-Perennou C Barbaroux C Servant N Vacher S Sigal-Zafrani B Importance of pre-analytical steps for transcriptome and RT-qPCR analyses in the context of the phase II randomised multicentre trial REMAGUS02 of neoadjuvant chemotherapy in breast cancer patients BMC Cancer 2011 11 215 10.1186/1471-2407-11-215 21631949 PMC3126791

[B20] Newman MEJ Modularity and community structure in networks Proc Natl Acad Sci U S A 2006 103 8577 8582 10.1073/pnas.0601602103 16723398 PMC1482622

[B21] Price ND Trent J El-Naggar AK Cogdell D Taylor E Hunt KK Pollock RE Hood L Shmulevich I Zhang W Highly accurate two-gene classifier for differentiating gastrointestinal stromal tumors and leiomyosarcomas Proc Natl Acad Sci U S A 2007 104 3414 3419 10.1073/pnas.0611373104 17360660 PMC1805517

[B22] Xu L Tan AC Winslow RL Geman D Merging microarray data from separate breast cancer studies provides a robust prognostic test BMC Bioinforma 2008 9 125 10.1186/1471-2105-9-125 PMC2409450 18304324

[B23] Ghaffari N Ivanov I Qian X Dougherty ER A CoD-based reduction algorithm for designing stationary control policies on Boolean networks Bioinformatics 2010 26 1556 1563 10.1093/bioinformatics/btq225 20421196

[B24] Taylor IW Linding R Warde-Farley D Liu Y Pesquita C Faria D Bull S Pawson T Morris Q Wrana JL Dynamic modularity in protein interaction networks predicts breast cancer outcome Nat Biotechnol 2009 27 199 204 10.1038/nbt.1522 19182785

[B25] Storey JD Tibshirani R Statistical significance for genomewide studies Proc Natl Acad Sci U S A 2003 100 9440 9445 10.1073/pnas.1530509100 12883005 PMC170937

[B26] Scherf U Ross DT Waltham M Smith LH Lee JK Tanabe L Kohn KW Reinhold WC Myers TG Andrews DT A gene expression database for the molecular pharmacology of cancer Nat Genet 2000 24 236 244 10.1038/73439 10700175

[B27] Ulitsky I Maron-Katz A Shavit S Sagir D Linhart C Elkon R Tanay A Sharan R Shiloh Y Shamir R Expander: from expression microarrays to networks and functions Nat Protoc 2010 5 303 322 10.1038/nprot.2009.230 20134430

[B28] Rodriguez-Viciana P Tetsu O Oda K Okada J Rauen K McCormick F Cancer targets in the Ras pathway Cold Spring Harb Symp Quant Biol 2005 70 461 467 10.1101/sqb.2005.70.044 16869784

[B29] Bartek J Lukas J Cell cycle. Order from destruction. Science 2001 294 66 67 10.1126/science.1066237 11588240

[B30] Momand J Wu HH Dasgupta G MDM2–master regulator of the p53 tumor suppressor protein Gene 2000 242 15 29 10.1016/S0378-1119(99)00487-4 10721693

[B31] Hershko T Chaussepied M Oren M Ginsberg D Novel link between E2F and p53: proapoptotic cofactors of p53 are transcriptionally upregulated by E2F Cell Death Differ 2005 12 377 383 10.1038/sj.cdd.4401575 15706352

[B32] Vaseva AV Moll UM The mitochondrial p53 pathway Biochim Biophys Acta 2009 1787 414 420 10.1016/j.bbabio.2008.10.005 19007744 PMC2819081

[B33] Regula KM Kirshenbaum LA p53 activates the mitochondrial death pathway and apoptosis of ventricular myocytes independent of de novo gene transcription J Mol Cell Cardiol 2001 33 1435 1445 10.1006/jmcc.2001.1405 11448132

[B34] Jane EP Premkumar DR Pollack IF AG490 influences UCN-01-induced cytotoxicity in glioma cells in a p53-dependent fashion, correlating with effects on BAX cleavage and BAD phosphorylation Cancer Lett 2007 257 36 46 10.1016/j.canlet.2007.06.020 17900801 PMC2055549

[B35] Jiang P Du W Wu M p53 and Bad: remote strangers become close friends Cell Res 2007 17 283 285 10.1038/cr.2007.19 17404594

[B36] Ueki K Fruman DA Brachmann SM Tseng YH Cantley LC Kahn CR Molecular balance between the regulatory and catalytic subunits of phosphoinositide 3-kinase regulates cell signaling and survival Mol Cell Biol 2002 22 965 977 10.1128/MCB.22.3.965-977.2002 11784871 PMC133541

[B37] Chen J Xu XM Underhill CB Yang S Wang L Chen Y Hong S Creswell K Zhang L Tachyplesin activates the classic complement pathway to kill tumor cells Cancer Res 2005 65 4614 4622 10.1158/0008-5472.CAN-04-2253 15930279

[B38] Feng Y Wan H Liu J Zhang R Ma Q Han B Xiang Y Che J Cao H Fei X Qiu W The angiotensin-converting enzyme 2 in tumor growth and tumor-associated angiogenesis in non-small cell lung cancer Oncol Rep 2010 23 941 948 20204277 10.3892/or_00000718

[B39] Lavie Y Cao H Bursten SL Giuliano AE Cabot MC Accumulation of glucosylceramides in multidrug-resistant cancer cells J Biol Chem 1996 271 19530 19536 10.1074/jbc.271.32.19530 8702646

[B40] Jones S Zhang X Parsons DW Lin JC Leary RJ Angenendt P Mankoo P Carter H Kamiyama H Jimeno A Core signaling pathways in human pancreatic cancers revealed by global genomic analyses Science 2008 321 1801 1806 10.1126/science.1164368 18772397 PMC2848990

[B41] Elston CW The assessment of histological differentiation in breast cancer Aust N Z J Surg 1984 54 11 15 10.1111/j.1445-2197.1984.tb06677.x 6586161

[B42] Acharya CR Hsu DS Anders CK Anguiano A Salter KH Walters KS Redman RC Tuchman SA Moylan CA Mukherjee S Gene expression signatures, clinicopathological features, and individualized therapy in breast cancer JAMA 2008 299 1574 1587 10.1001/jama.299.13.1574 18387932

[B43] Welcker M Clurman BE FBW7 ubiquitin ligase: a tumour suppressor at the crossroads of cell division, growth and differentiation Nat Rev Cancer 2008 8 83 93 10.1038/nrc2290 18094723

[B44] Steeg PS Abrams JS Cancer prognostics: past, present and p27 Nat Med 1997 3 152 154 10.1038/nm0297-152 9018230

